# Interpretable machine learning model to predict surgical difficulty in laparoscopic resection for rectal cancer

**DOI:** 10.3389/fonc.2024.1337219

**Published:** 2024-02-06

**Authors:** Miao Yu, Zihan Yuan, Ruijie Li, Bo Shi, Daiwei Wan, Xiaoqiang Dong

**Affiliations:** Department of General Surgery, The First Affiliated Hospital of Soochow University, Suzhou, China

**Keywords:** rectal cancer, pelvimetry, surgical difficulty, prediction model, machine learning, Shapley additive explanations

## Abstract

**Background:**

Laparoscopic total mesorectal excision (LaTME) is standard surgical methods for rectal cancer, and LaTME operation is a challenging procedure. This study is intended to use machine learning to develop and validate prediction models for surgical difficulty of LaTME in patients with rectal cancer and compare these models’ performance.

**Methods:**

We retrospectively collected the preoperative clinical and MRI pelvimetry parameter of rectal cancer patients who underwent laparoscopic total mesorectal resection from 2017 to 2022. The difficulty of LaTME was defined according to the scoring criteria reported by Escal. Patients were randomly divided into training group (80%) and test group (20%). We selected independent influencing features using the least absolute shrinkage and selection operator (LASSO) and multivariate logistic regression method. Adopt synthetic minority oversampling technique (SMOTE) to alleviate the class imbalance problem. Six machine learning model were developed: light gradient boosting machine (LGBM); categorical boosting (CatBoost); extreme gradient boost (XGBoost), logistic regression (LR); random forests (RF); multilayer perceptron (MLP). The area under receiver operating characteristic curve (AUROC), accuracy, sensitivity, specificity and F1 score were used to evaluate the performance of the model. The Shapley Additive Explanations (SHAP) analysis provided interpretation for the best machine learning model. Further decision curve analysis (DCA) was used to evaluate the clinical manifestations of the model.

**Results:**

A total of 626 patients were included. LASSO regression analysis shows that tumor height, prognostic nutrition index (PNI), pelvic inlet, pelvic outlet, sacrococcygeal distance, mesorectal fat area and angle 5 (the angle between the apex of the sacral angle and the lower edge of the pubic bone) are the predictor variables of the machine learning model. In addition, the correlation heatmap shows that there is no significant correlation between these seven variables. When predicting the difficulty of LaTME surgery, the XGBoost model performed best among the six machine learning models (AUROC=0.855). Based on the decision curve analysis (DCA) results, the XGBoost model is also superior, and feature importance analysis shows that tumor height is the most important variable among the seven factors.

**Conclusions:**

This study developed an XGBoost model to predict the difficulty of LaTME surgery. This model can help clinicians quickly and accurately predict the difficulty of surgery and adopt individualized surgical methods.

## 1 Introduction

According to the latest statistics, the incidence of colorectal cancer in the world has ranked the third among malignant tumors, and the mortality rate has ranked second, among which the incidence of rectal cancer ranks eighth (1). To a large extent, it has become a public health problem threatening human health. Rectal cancer has a rate approaching that of colon cancer and is a heavy health burden in the world. Since the introduction of total mesorectal excision (TME) in the 1980s by Heald (2)et al., the quality of TME directly affects the recurrence of local tumor and the prognosis of patients. So TME has become the gold standard for surgical treatment of rectal cancer. In the past two decades, with the development of minimally invasive surgery, laparoscopic surgery can be combined with classical surgery to achieve minimally invasive. Compared with open surgery, laparoscopic total mesorectal excision (LaTME) has the advantages of less invasive nature, faster recovery and better visualization of surgical field (3, 4), so it has become one of the main surgical methods for rectal cancer. Due to the fixed bony structure of pelvis and the limited space for pelvic surgery, it is hardto keep a clear surgical field of vision, identify accurate anatomical structures and perform accurate rectal resection (5). So, In rectal cancer, especially in deep and narrow pelvises, LaTME can be technically challenging. However, open surgery can better expose the surgical field of vision and accurately touch the extent of the tumor. Also, emerging techniques such as transanal total mesorectal excision (TaTME) and robotic surgery may help overcome the difficulties encountered during LaTME (6–8). Therefore, early identification of difficult LaTME surgery is necessary. Magnetic resonance imaging (MRI) has been widely used in routine (9–11) preoperative evaluation in the diagnosis and treatment of rectal cancer. It can not only clearly show the pelvic anatomy and soft tissue structure around the rectum, but also evaluate the depth of tumor invasion and suspected lymphatic metastasis around the mesorectum. A recent meta-analysis (12) shows that pelvic measurements based on MRI pelvic measurements can predict the difficulty of TME surgery. Therefore, MRI is a very useful tool in rectal cancer.

In recent years, artificial intelligence has developed rapidly, especially machine learning has been widely used in many medical fields because of its excellent performance (13, 14). Currently, there are few reports on machine learning models predicting the difficulty of LaTME surgery. In clinical practice, only some traditional statistical tools like nomograms that predict surgical difficulty (15, 16).Therefore, the purpose of this study is to explore the risk factors affecting the difficulty of LaTME surgery, to develop a preoperative, non-invasive and quantitative accurate strategy, and to establish an interpretable machine learning model to help clinicians choose appropriate surgical approach.

## 2 Materials and methods

### 2.1 Study design and subjects

This retrospective study collected the data of rectal cancer patients undergoing LaTME at The First Affiliated Hospital of Soochow University from 2017 to 2022. Patient inclusion criteria were as follows (1): colonoscopy showed that the distance from the lower margin of the tumor to the anal margin was less than 15cm, and it was confirmed as rectal adenocarcinoma by biopsy (2), preoperative rectal MRI scan was performed in our hospital within 15 days before surgical resection (3), execute LaTME strictly according to the principle of TME.

The exclusion criteria were as follows (1): without rectal MRI in our hospital (2), multiple primary cancer, secondary tumor, recurrence, distant metastasis (3), underwent abdominoperineal resection (APR) or other surgeries (e.g., Hartmann’s procedure, emergency surgery, palliative surgery, multivisceral resection, or lateral pelvic lymph node dissection) (4), history of previous pelvic surgery (5), patients receiving neoadjuvant therapy. Moreover, all rectal cancer operations are performed by an experienced laparoscopic surgery team (the chief surgeon has more than ten years of experience in laparoscopic surgery) to follow the TME procedure. Some patients underwent ileostomy at the same time of resection. In order to reduce the impact of this operation, the operation time of these patients was recorded as the initial time minus 15 minutes (17). When the entire operation cannot be completed by laparoscopy, it should be changed to acombined approach (transabdominal and transanal surgery).

**Figure 1:**
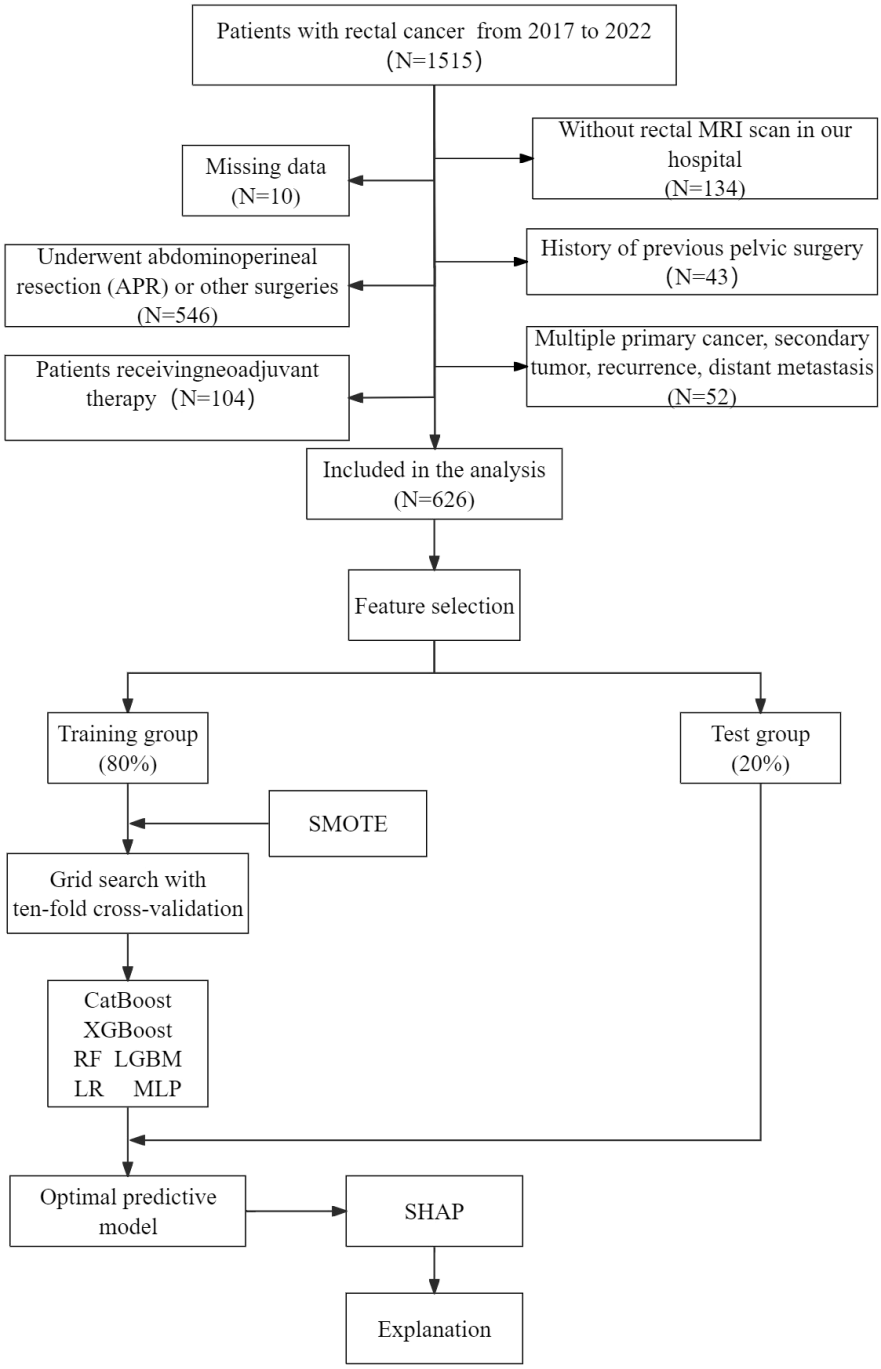
Flowchart of patient selection and machine learning model development process. LR, logistic regression; LGBM, light gradient boosting machine; CatBoost, categorical boosting; MLP, multilayer perceptron; RF, random forests; XGBoost, extreme gradient boost; SMOTE, synthetic minority oversampling technique; SHAP, shape additive explanation.

**Figure 1** shows a flowchart outlining patient enrollment and study design. Finally, 626 rectal cancer patients who received LaTME were randomly divided into training group (80%) and test group (20%). The training group uses machine learning algorithm to train and optimize the models, and the test group is used to test the prediction performance of these models.

### 2.2 Definition of surgical difficulty

We evaluate the difficulty of LaTME by intraoperative and postoperative parameters. Because there are many differences between eastern and western patients, we modify the standard of surgical difficulty proposed by Escal (18) et al. Surgical difficulty score: duration of surgery > 240 min (3 points),blood loss >200 ml (1 point), conversion to laparotomy (3 points), postoperative complications (grade II and III) (1 point), use of transanal dissection (2 points), and postoperative hospital stay >12 days (2 points). And the patients were divided into two groups: low surgical difficulty group (<6 points) and high surgical difficulty group (≥ 6 points). The postoperative complications was graded according to the Clavien–Dindo classification (19). Grade II: Medical treatment is required, including blood transfusion or total parenteral nutrition. Grade III: surgical, endoscopic, or radiological intervention is required.

### 2.3 MRI pelvimetry and other variables

**Figure 2:**
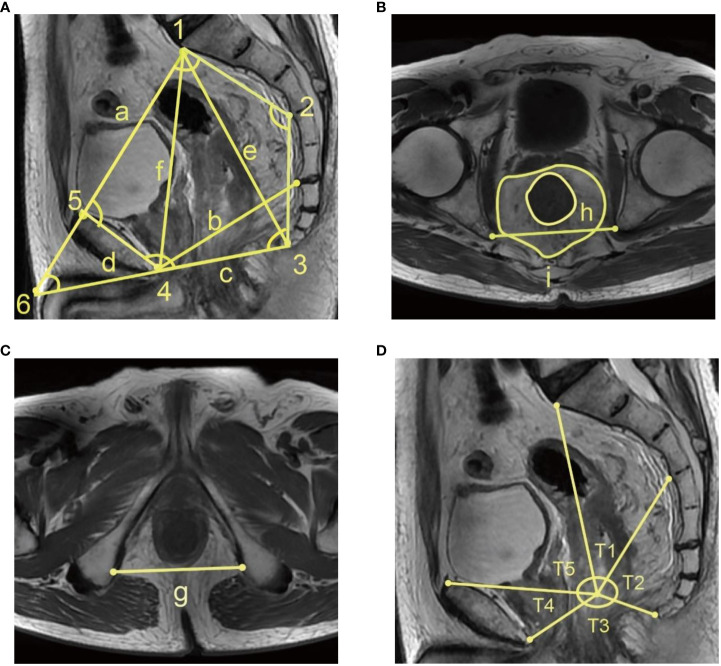
MRI T2 weighted image pelvimetry **(A)** Median sagittal position: (a) pelvic inlet, (b) middle pelvis, (c) pelvic outlet (d) Pubic symphysis height, (e) sacrococcygeal distance, (f) Internal diameter of sacrum and pubis (1), Angle 1 (2), Angle 2 (3), Angle 3 (4), Angle 4 (5), Angle 5 (6), Sacrococcygeal–pubic angle **(B)** Ischiatic tuberosity horizontal transverse position:(g) Intertuberous distance **(C)** Fifth sacral vertebral tip horizontal transverse position: (h) mesorectal fat area, (i) Interischial distance **(D)** Median sagittal position: (T1) Angle T1, (T2) Angle T2, (T3) Angle T3, (T3) Angle T3, (T4) Angle T4, (T5) Angle T5.

All rectal cancer patients underwent abdominal pelvic 3.0T MRI examination within 15 days before surgery. The publicly available software (3DSlicer, version 5.2.2) funded by the National Institutes of Health was used for pelvic measurement and analysis (20). T2-weighted imaging (T2WI) was used to measure pelvic measurements, and all pelvic MR images were reviewed retrospectively by an observer blinded to the patients’ clinicopathological information. Specific measurement parameters are shown in **Figure 2**. The measurements obtained are as follows (21, 22):

1. Pelvic inlet: the distance from the median surface of the superior symphysis pubis to the promontory;
2. Middle pelvis: the distance between the midpoint of the lower margin of the symphysis pubis and the midpoint of the anterior edge of the sacrococcygeal junction;
3. Pelvic outlet: the distance from the lower margin of the symphysis pubis to the coccyx;
4. Interischial distance: the distance between the sciatic spines on both sides.
5. Intertuberous distance: the distance between the innermost points of the ischial tuberosities;
6. Pubic symphysis height: the distance between the upper and lower margins of the symphysis pubis;
7. Sacrococcygeal distance: the distance from the promontory to the tip of the tailbone;
8. Internal diameter of sacrum and pubis: the distance from the promontory to the inferior margin of pubis;
9. Mesorectal fat area: the mesentery and fatty area surrounding the rectum at the tip of the fifth sacral vertebra;
10. Sacrococcygeal–pubic angle: the angle between an extension of the line forming the anteroposterior diameter of the pelvic inlet and that of the anteroposterior diameter of the pelvic outlet the angle between the extension of the anteroposterior diameter line of the pelvic inlet and the extension of the anteroposterior diameter line of the pelvic outlet;
11. Angle 1: the angle between the pubic symphysis, the upper boundary of the promontory and the middle of the S3 vertebral body;
12. Angle 2:the angle between the cape, the middle of the S3 vertebrae, and the tailbone;
13. Angle 3: the angle between the middle of the S3 vertebral body, the coccyx and the lower edge of the pubic symphysis;
14. Angle 4: the angle between the coccyx, the upper and lower borders of the pubic symphysis;
15. Angle 5: the angle between the superior and inferior border lines of the pubic symphysis and the midpoint of the superior border of the pubic symphysis and the line between the sacral promontory;
16. Angle T1: the angle between the apex of the sacral angle and the lower edge of the third sacrum;
17. Angle T2: the angle between the lower margin of the tubercle of the third sacrum and the apex of the coccyx;
18. Angle T3: the angle between the apex of the tailbone and the lower margin of the pubis;
19. Angle T4: the angle between the upper and lower borders of the pubic symphysis with the lower border of the tumor as the vertex;
20. Angle T5: the angle between the superior margin of the pubis and the apex of the promontory.

In addition, we obtained the baseline characteristics of the patients from the medical record: age, gender, BMI, albumin, globulin, lymphocyte count and tumor height. Among them, hematology nutritional indicators are added (23), and the calculation is as follows: albumin to globulin ratio (AGR)=albumin/globulin, prognostic nutrition index (PNI)=serum albumin (g/L) +5*lymphocyte count (10^9^/L). Blood samples were collected within one week before surgery. We also collected the pathological stages of the patients’ surgical specimens, and the tumors were staged according to the 8th tumor-node-metastasis (TNM) classification of the National Comprehensive Cancer Network (NCCN) and American Joint Committee on Cancer (AJCC) (24).

### 2.4 Development and validation of prediction models

In order to ensure the simplicity of our model, T-test, Mann-Whitney U test and Chi-square test were carried out to screen the variables with statistical differences between the high and low surgical difficulty groups. Then we use the LASSO regression of 10-fold cross-validation to reduce the dimension. Finally, the variables with non-zero coefficients are analyzed by multivariable logistics regression to screen independent risk factors to build a machine learning model. In our study, there was a serious imbalance between the low surgical difficulty group and high surgical difficulty group. Unbalanced data sets are frequently encountered in medical research due to the disproportionate number of non-patients compared to patients, leading to diminished predictive performance (25). The Synthetic Minority Oversampling Technique (SMOTE) is an efficient algorithm for addressing class imbalances (26), employing k-neighbor synthesis to focus on a limited number of classes and achieve a balanced dataset (27), which has demonstrated commendable efficacy in disease detection. So, we use the SMOTE to solve the problem of data imbalance and reduce the over-fitting of the model. SMOTE was only applied to our training group, and we did not oversample the test set, thus maintaining the natural frequency of results.

We use the data set after SMOTE to build six machine learning prediction models, including light gradient boosting machine (LGBM); categorical boosting (CatBoost); extreme gradient boost (XGBoost), logistic regression (LR); random forests (RF); multilayer perceptron (MLP). The subjects were randomly divided into training group (80%) and test group (20%). The training group was used for model development and hyperparameter tuning, the test group was used for model evaluation verification, and we use grid search with ten-fold cross-validation to find and determine optimal parameters for machine learning algorithms. The grid search algorithm systematically arranges and combines all possible parameter values, subsequently substituting the results of each combination into the model training process. The objective is to identify the optimal parameter combination from the exhaustive set of possibilities. Use discrimination and calibration to validate the model’s predictive ability. The area under the receiver operating characteristic curve (AUROC) represents a measure of discrimination, and the performance of a model is evaluated through accuracy, sensitivity, specificity and F1 score. The Brier score and calibration curve were employed for model calibration. The Brier score represents the average squared deviation between the predicted outcome probability and the true label. A lower Brier score indicates superior model performance. The clinical effective rate and net benefit were evaluated by decision curve analysis (DCA). The Shapley Additive Interpretation (SHAP) is employed to directly elucidate the impacts of significant variables on the model. SHAP, a model interpretation technique grounded in cooperative game theory (28), has recently demonstrated its efficacy in explicating diverse machine learning models (29–31). Specifically, SHAP assigns each feature with a Shapley value by classifying the model’s output value. Intuitively, estimating the Shapley value for each feature enables us to explicate its contribution to the outcome. The Shapley value accurately reflects the influence of a feature in each sample and facilitates a deeper understanding of whether it acts as a protective or risk factor for the model. The SHAP summary chart is generated from the Shapley value, the importance of the features is ranked, and the SHAP force plot is constructed to analyze and interpret the prediction results of a single sample.

### 2.5 Statistical analysis

All statistical analysis was carried out with IBM SPSS (version 26.0), R (version 4.2.3) and Python(version 3.10.0). The Shapiro-Wilk test was utilized to assess the normality of the data. Continuous data conforming to a normal distribution were presented as mean and standard deviation (SD), while continuous data deviating from a normal distribution were expressed as median and interquartile range (IQR). Student’s t-test was employed for comparing continuous data following a normal distribution, whereas Mann-Whitney U test was used for comparing non-normal distribution data. Disaggregated data were reported as frequency (percentage), and comparisons between the two groups were conducted using the χ^2^ test or Fisher’s exact test (if the theoretical frequency T < 5). A p-value less than 0.05 in bilateral testing was considered statistically significant.

## 3 Results

### 3.1 Patient characteristics and surgical outcomes

**Table 1** shows the clinical features and MRI pelvimetry of all participants. A total of 626 patients were included in this study, of which the median age was 64 (56–71) years old. The majority of the patients were male, accounting for 59.7% of the total. The median height of tumor was 9 (7 ~ 12) cm. Among the indicators related to the surgical difficulty, the probability that the median time of operation, blood loss and postoperative hospital stay were 198.5 (160.0, 240.5) min, 100 (50, 200) ml and 10 (8, 12) days. Use of transanal dissection, conversion to open procedure and morbidity (grade II and III) were 21.6%, 27.3% and 29.7%, respectively. Other indicators are shown in **Table 1**. Compared with the patients with low surgical difficulty, the patients in the high surgical difficulty group had lower tumor height, lower PNI, shorter pelvic inlet, pelvic outlet, longer sacrococcygeal distance, more mesorectal fat area and larger angle 5 and sacrococcygeal-pubic angle. However, preliminary analysis showed that there was no significant difference in gender, age, BMI, AGR, pathological T stage, pathological N stage, pathological TNM stage, middle pelvis, interischial distance, Intertuberous distance, pubic symphysis height, internal diameter of sacrum and pubis, angle 1, angle 2, angle 3, angle4 and tumor related angle between the two groups.

**Table 1:** Clinical features and MRI pelvimetry of all participants in different groups.

Variables	Overall (N=626)	Low surgical Difficulty group (N=516)	High surgical Difficulty group (N=110)	*P*
Baseline characteristics				
Gender (%)				0.221
Male	374 (59.7%)	314 (60.85%)	60 (54.55%)	
Female	252 (40.3%)	202 (39.15%)	50 (45.45%)	
Age (median [IQR], year)	64 (56, 71)	64 (56,71)	64 (58,71)	0.647
BMI (median [IQR], kg/m²)	23.63 (21.54,25.52)	23.66 (21.61,24.02)	23.44 (21.06,25.27)	0.316
Tumor height (median [IQR], cm)	9 (7,12)	10 (7,12)	7 (5,10)	**<0.001**
Hematology nutritional indicators				
AGR (median [IQR])	1.52 (1.37,1.69)	1.51 (1.37,1.69)	1.59 (1.38,1.73)	0.248
PNI (mean [SD])	49.5 (5.22)	49.81 (4.86)	48.00 (6.47)	**0.006**
Pathological stage				
Pathological T stage (%)				0.115
T1	7 (1.12%)	5 (0.97%)	2 (1.82%)	
T2	100 (15.97%)	75 (14.53%)	25 (22.73%)	
T3	469 (74.92%)	396 (76.75%)	73 (66.36%)	
T4	50 (7.99)	40 (7.75%)	10 (9.09%)	
Pathological N stage (%)				0.115
N0	320 (51.12%)	255 (49.42%)	65 (59.09%)	
N1	168 (26.84%)	145 (28.10%)	23 (20.91%)	
N2	138 (22.04)	116 (22.48%)	22 (20%)	
Pathological TNM stage (%)				0.838
I	79 (12.62%)	61 (11.82%)	18 (16.36%)	
II	247 (39.46%)	209 (40.50%)	38 (34.55%)	
III	300 (47.92%)	246 (47.67%)	54 (49.09%)	
MRI pelvimetry				
Pelvic inlet (mean [SD], cm)	11.74 (1.07)	11.82 (1.05)	11.36 (1.08)	**<0.001**
Middle pelvis (mean [SD], cm)	12.55 (0.99)	12.57 (0.97)	12.49 (1.03)	0.444
Pelvic outlet (mean [SD], cm)	8.78 (0.89)	8.83 (0.91)	8.53 (0.77)	**0.001**
Interischial distance (median [IQR], cm)	9.74 (8.92,10.66)	9.74 (8.98,10.60)	9.74 (8.70,10.80)	0.738
Intertuberous distance (median [IQR], cm)	9.97 (8.79,11.20)	9.98 (8.81,11.09)	9.94 (8.68,11.39)	0.904
Pubic symphysis height (median [IQR], cm)	4.74 (4.27,5.15)	4.71 (4.26, 5.14)	4.80 (4.28,5.18)	0.388
Sacrococcygeal distance (median [IQR], cm)	12.60 (11.66,13.37)	12.50 (11.58,13.26)	12.98 (11.99,13.66)	**<0.001**
Internal diameter of sacrum and pubis (mean [SD], cm)	12.84 (1.14)	12.81 (1.13)	12.96 (1.15)	0.222
Mesorectal fat area (median [IQR], cm²)	16.65 (11.57,21.88)	16.3 (10.97,21.40)	18.08 (13.36,23.60)	**0.001**
Angle 1 (median [IQR], °)	116.1 (107.0,124.2)	116.1 (107.6,124.4)	116.8 (104.4,124.0)	0.463
Angle 2 (mean [SD], °)	108.3 (10.9)	108.4 (10.9)	107.6 (11.2)	0.439
Angle 3 (median [IQR], °)	127.2 (122.2,132.8)	127.3 (122.7,133.2)	126.8 (120.5,131.9)	0.086
Angle 4 (median [IQR], °)	89.1 (82.6,96.4)	89.0 (82.2,96.0)	90 (83.2,97.7)	0.486
Angle 5 (median [IQR], °)	98.5 (93.7,103.8)	97.7 (93.5,103)	101 (95.8,109.5)	**<0.001**
Sacrococcygeal–pubic angle(median [IQR], °)	46.9 (41.0,52.5)	46.4 (40.8,51.6)	49.0 (41.9,56.0)	**0.008**
Angle T1 (median [IQR], °)	53.9 (46.8,68.5)	54.2 (47.0,68.6)	53.0 (44.8,68.0)	0.342
Angle T2 (median [IQR], °)	79.9 (59.0,101.6)	81.0 (60.9,101.9)	72.8 (51.1,100.6)	0.089
Angle T3 (median [IQR], °)	112.1 (80.1,143.2)	110.4 (80.8,141.0)	114.8 (78.0,150.5)	0.380
Angle T4 (median [IQR], °)	27.0 (23.0,31.0)	27.0 (23.1,31.2)	26.0 (22.2,30.0)	0.134
Angle T5 (median [IQR], °)	72.6 (66.7,80.0)	72.6 (67.0,79.9)	72.6 (64.5,80.9)	0.690
Surgical difficulty				
Duration of surgery (median [IQR], min)	198.5 (160.0,240.5)	187 (153,221.5)	260 (222.3,291.5)	**<0.001**
Blood loss (median [IQR], ml)	100 (50,200)	100 (50,200)	150 (100,200)	**<0.001**
Postoperative hospital stays (median [IQR], day)	10 (8,12)	9 (8,11)	13 (9,16.3)	**<0.001**
Morbidity (grade II and III) (yes/no, %)	186/440 (29.7/70.3)	131/385 (25.4/74.6)	55/55 (50/50)	**<0.001**
Use of transanal dissection (yes/no, %)	135/491 (21.6/78.4)	74/442 (14.3/85.7)	61/49 (55.5/44.5)	**<0.001**
Conversion to open procedure (yes/no, %)	171/455 (27.3/72.7)	102/414 (19.8/80.2)	69/41 (62.7/37.3)	**<0.001**

IQR, interquartile range; SD, standard deviation; BMI, body mass index; AGR, albumin to globulin ratio; PNI, prognostic nutrition index; The bold values P <0.05.

### 3.2 The relationship between clinicopathological factors and the definition of surgical difficulty

The comparison of clinicopathological parameters of rectal cancer patients with six definitions of surgical difficulty is shown in **Supplementary Table 1**. Intertuberous distance had an association with duration of surgery, an association mesorectal fat area between and more estimated blood loss was found BMI PNI pathological T stage internal diameter of sacrum and pubis had an association with conversion to open procedure, angle T1 angle T2 angle T3 had associations with morbidity and use of transanal dissection, and there was an association of angle T4 with postoperative hospital stay. Also pubic symphysis height angle 3 angle 5 can influence the morbidity, and tumor height pathological TNM stageIII can affect use of transanal dissection. All the above associations were statistically significant (all p < 0.05).

### 3.3 Feature selection

**Figure 3:**
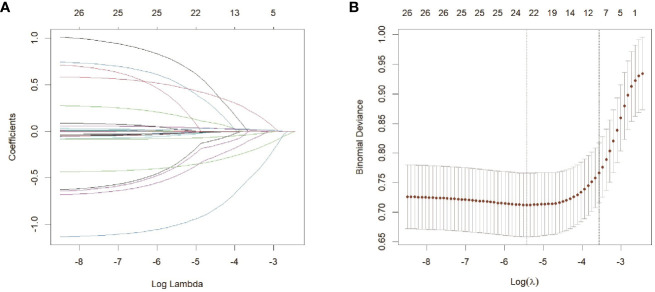
Feature selection based on LASSO regression analysis**(A)** LASSO coefficient profiles of the 26 variables. **(B)** Selection of the optimal penalization coefficient lambda in the LASSO model used ten-fold cross validation based on minimum criteria. The partial likelihood deviance is plotted against log (lambda), where lambda is the tuning parameter. Red dots indicate average deviance values for each model with a given lambda, and partial likelihood deviance values are shown, with error bars representing SE. Dotted vertical lines were drawn at the optimal values by using the minimum criteria and the 1 SE of the minimum criteria (the 1-SE criteria).

**Figure 4:**
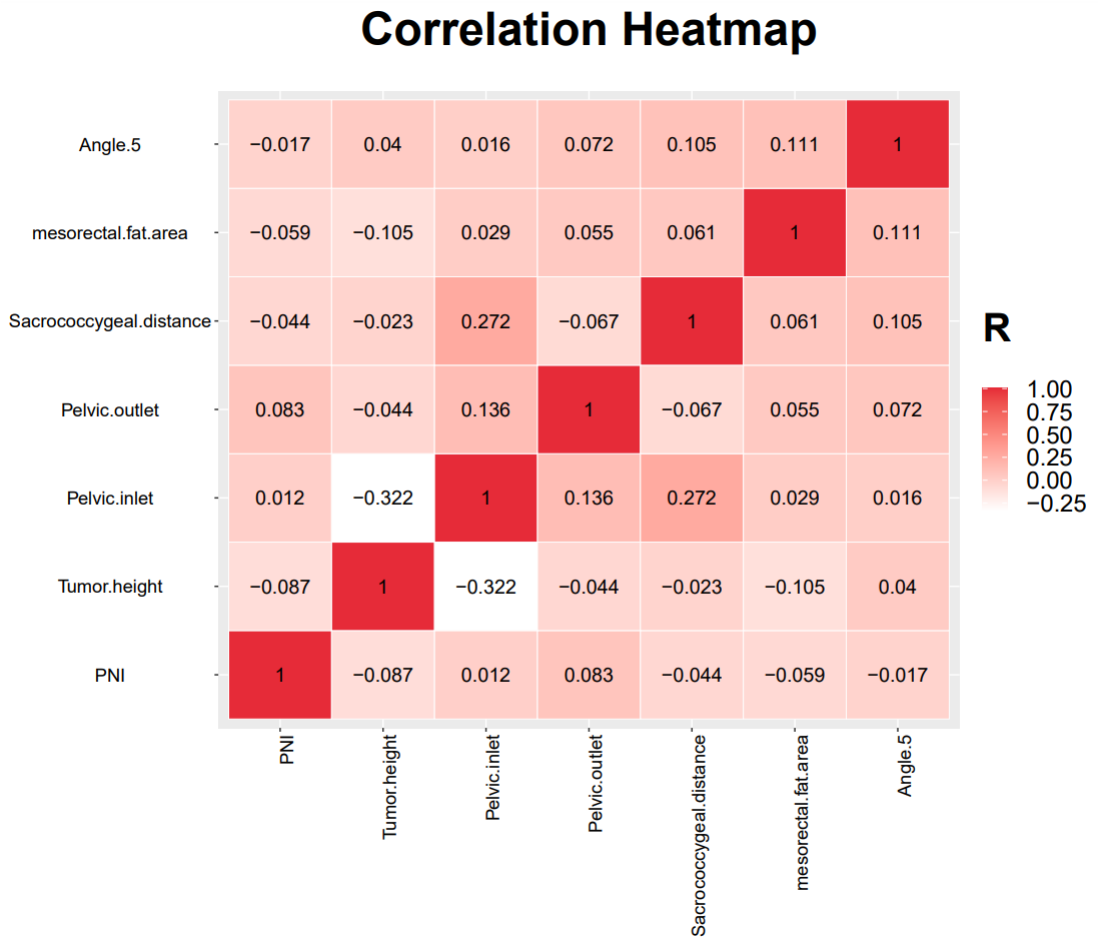
Results of the correlation heatmap between all variables.

LASSO can compress variable coefficients to prevent over-merging to solve serious collinearity problems (32). We use LASSO regression analysis and ten-fold cross-validation to filter variables. Use 1 standard error’s lambda to select seven variables (**Figure 3**), including tumor height, PNI, pelvic inlet, pelvic outlet, sacrococcygeal distance, mesorectal fat area and angle 5. In order to further control the influence of confounding factors, the above seven independent variables were analyzed by multivariate logistic regression analysis (**Table 2**). We found that the above seven variables are independent influencing factors for the difficulty of LaTME surgery. The correlation heatmap (**Figure 4**) results show that the correlations between variables are all less than 0.4, there is no significant correlation between variables, and there is no multicollinearity. Finally, tumor height, PNI, pelvic inlet, pelvic outlet, sacrococcygeal distance, mesorectal fat area and angle 5 were selected to be included in the machine learning model.

**Table 2:** Based on the coefficients and Lambda.1se values of the LASSO regression, multivariable logistics regression to validate the validity of each variable.

Variables	LASSO regression		Multivariable logistics regression	
	Coefficients	Lambda.1se	OR (95%CI)	*P*
Tumor height	-0.20860369	0.02837605	0.656 (0.588-0.733)	<0.001
PNI	-0.03522676		0.916 (0.874-0.960)	<0.001
Pelvic inlet	-0.48595986		0.351 (0.264-0.467)	<0.001
Pelvic outlet	-0.09046064		0.730 (0.547-0.975)	0.033
Sacrococcygeal distance	0.2097511		1.686 (1.346-2.112)	<0.001
Mesorectal fat area	0.01126133		1.041 (1.003-1.081)	0.035
Angle 5	0.02102902		1.043 (1.021-1.064)	<0.001

Coefficients, coefficients of each variable in LASSO regression; Lambda.1se, among all lambda values, the lambda value of the simplest model within a variance of the mean value of the minimum target parameter is obtained; OR, odds ratio; CI; confidence interval; PNI, prognostic nutrition index.

### 3.4 Performance of the machine learning model and model interpretability

**Figure 5:**
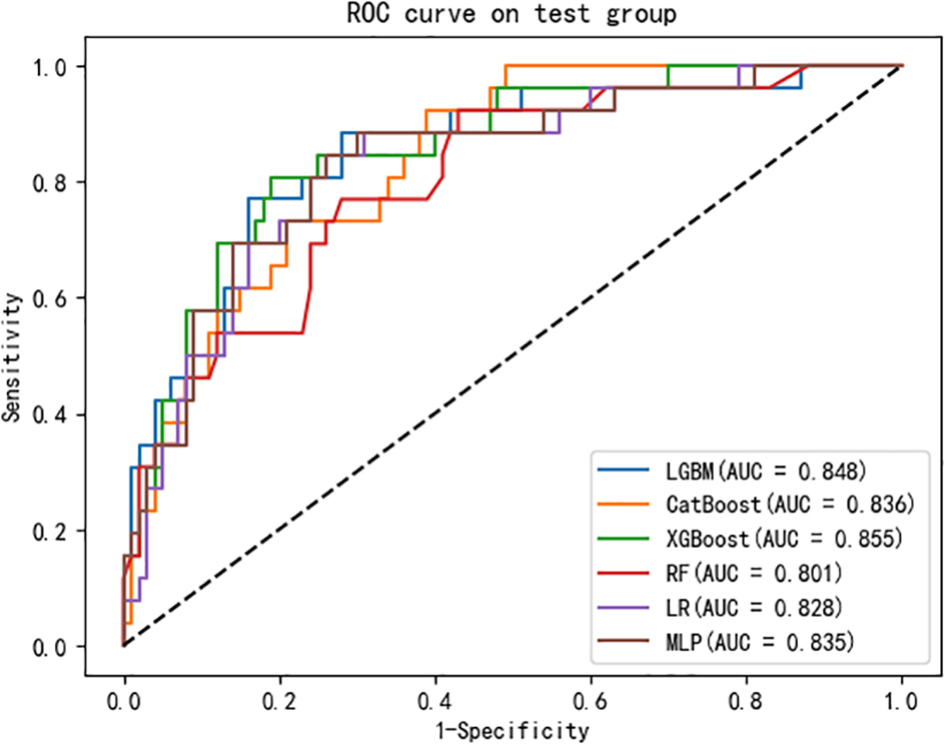
Evaluation of the six machine learning models based on the AUC of the ROC curve in validation set. AUC, area under the curve; ROC, receiver operating characteristic.

**Figure 6:**
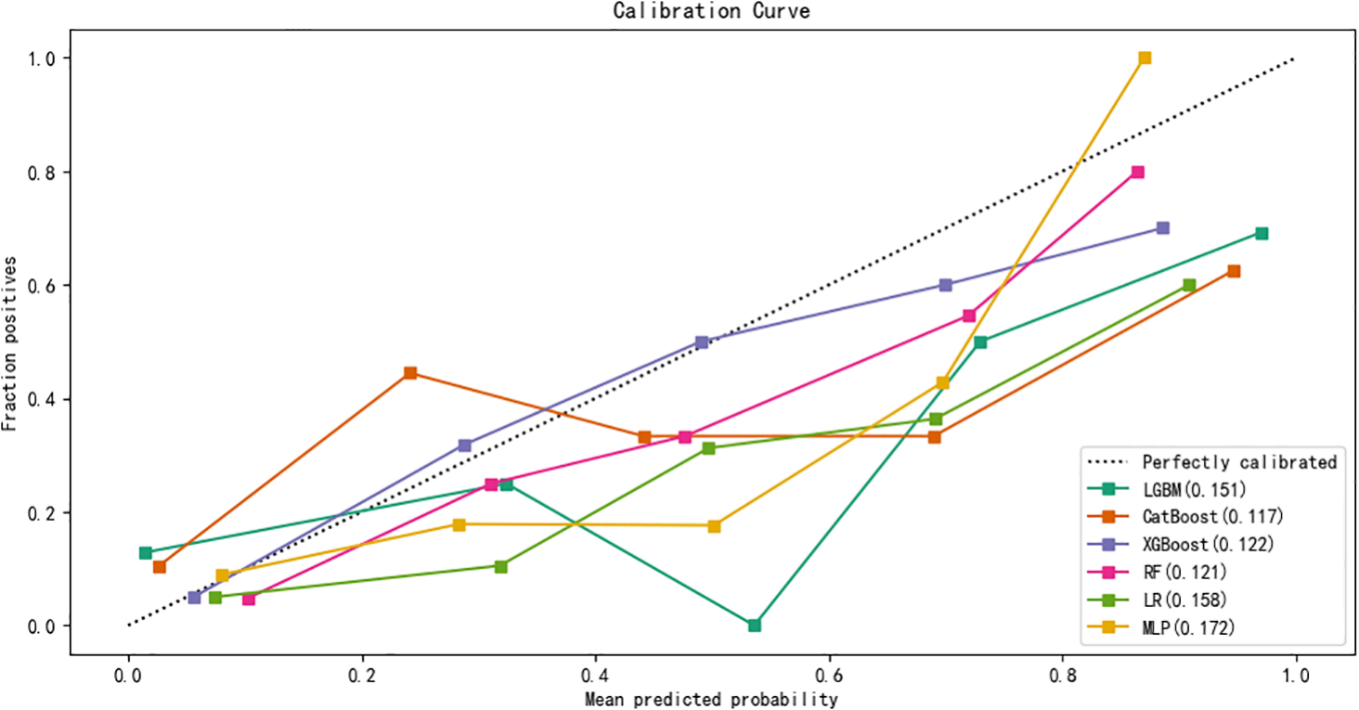
Calibration curves of five machine learning models in the validation set.

**Figure 7:**
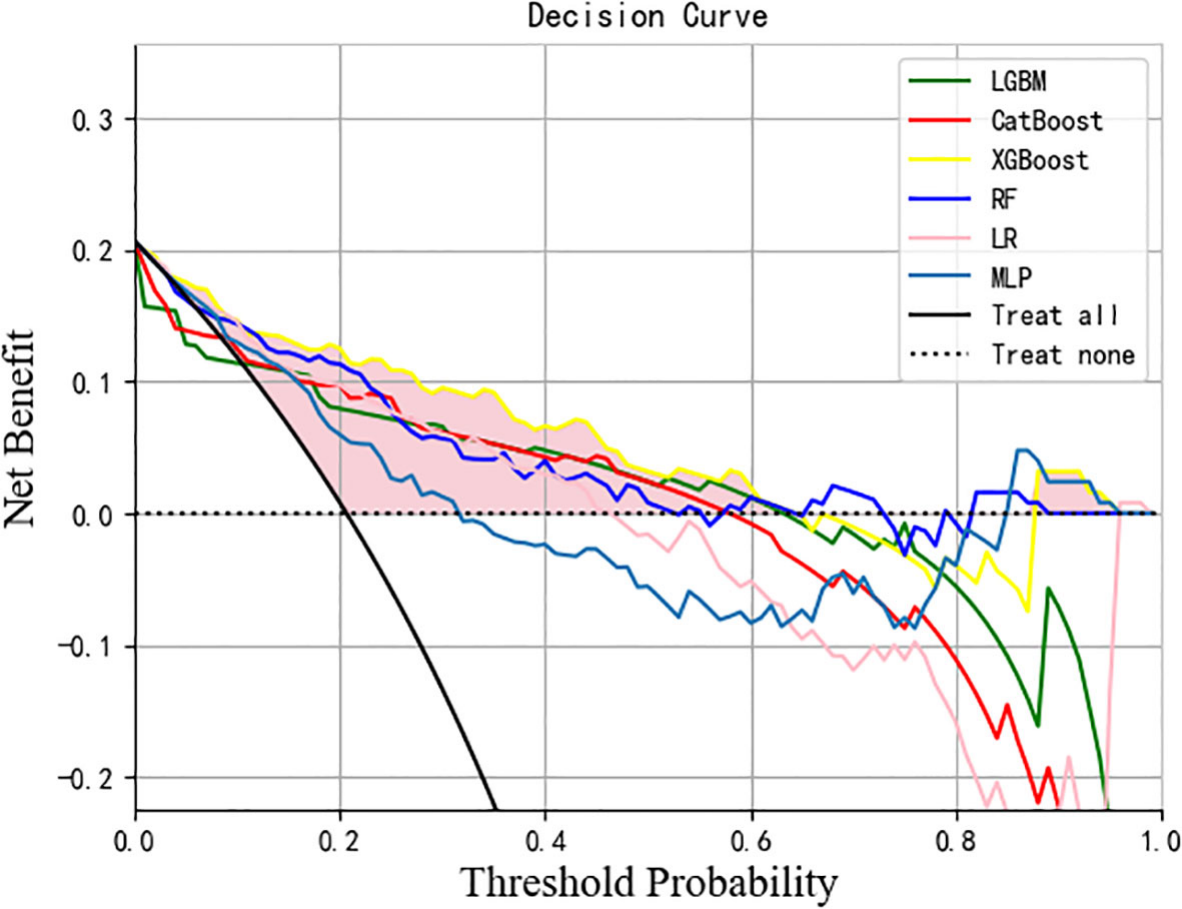
DCA analysis was performed to evaluate the clinical usefulness. The y-axis indicated the net benefit; the x-axis indicated the threshold probability. The solid yellow line shows the net benefit rate of the XGBoost forecast model. Within a certain threshold range, the XGBoost model has a higher net benefit. DCA, Decision curve analysis.

The data were randomly divided into a training group (80%, N = 500) and a test group (20%, N = 126) as shown in **Supplementary Table 2**. There was no statistical difference in most predictive variables between the training group and test group. In the training group, there were 84 high-difficulty operations and 416 low-difficulty operations. In the test group, 26 patients underwent high-difficulty surgery and 100 patients underwent low-difficulty surgery. There is a serious imbalance. After resampling the training set, SMOTE 416 cases of high difficulty and 416 cases of low difficulty. The seven variables after feature selection are used as predictor variables to build different prediction models. The optimization model was ten-fold cross-validation on the training data set, and the mesh search algorithm was used to find the optimal parameters of the machine learning algorithm. The best parameters of each model are shown in **Supplementary Table 3**. The ability to validate previously established predictive models with test queues. The results of AUROC, accuracy, sensitivity, specificity, F1 score and Brier score in the test set are shown in **Table 3**. From the overall performance of each model, in terms of discrimination, as shown in **Figure 5**, the AUROC of LGBM model is 0.848, the AUROC of CatBoost model is 0.836, the AUROC of XGBoost model is 0.855, the AUROC of RF model is 0.801, the AUROC of LR model is 0.828, and AUROC of MLP model is 0.835. The corresponding Brier score is 0.151, 0.117, 0.122, 0.121, 0.158 and 0.172 as shown in **Figure 6**. Decision curve analysis (DCA) showed that XGBoost model showed better clinical than other models before the threshold probabilities of 0.6(**Figure 7**). The XGBoost algorithm is selected to construct the prediction model after a comprehensive comparison.

**Table 3:** Performance of predictive models generated by five machine learning models.

Model	AUROC	Accuracy	Sensitivity	Specificity	F1 score
LGBM	0.848	0.841	0.500	0.93	0.565
XGBoost	0.855	0.841	0.538	0.92	0.583
CatBoost	0.84	0.817	0.423	0.92	0.489
LR	0.828	0.770	0.731	0.78	0.567
RF	0.820	0.817	0.423	0.92	0.489
MLP	0.835	0.802	0.692	0.83	0.590

**Figure 8:**
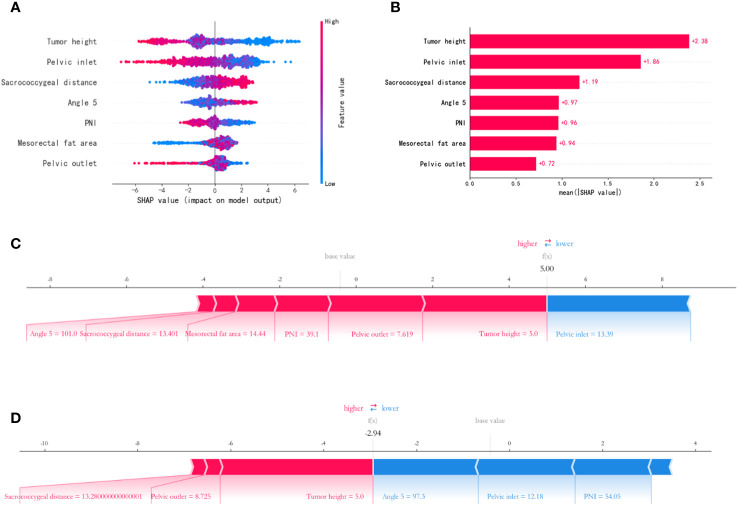
Feature importance SHAP summary chart and bar chart. **(A)** The left dot plot represents the direction of contribution of each value of each variable, with red representing larger values and blue representing lower values of each variable. **(B)** The bars on the right represent the importance of the variables and their overall contribution to the model predictions. **(C, D)** SHAP scores explain the predicted risk of osteoporosis in two subjects.

By calculating the contribution of each variable to the prediction, the results of the XGBoost model are interpreted using SHAP. The SHAP summary chart and importance matrix diagram of the XGBoost model is shown in **Figure 7**. The SHAP summary plot (**Figure 8A**) is based on estimates, with each patient having a data point for each feature. Red indicates higher values while blue represents lower values of the same. The horizontal axis shows the SHAP value, and larger shapes indicate features that have a higher predictive value for surgical difficulty in a given sample. The importance bar chart (**Figure 8B**) displays the significance of each variable in predicting surgery difficulty. To sum up, the features in descending order of importance are: tumor height, pelvic inlet, sacrococcygeal distance, angle 5, PNI, mesorectal fat area and pelvic outlet.

Applying predictive model SHAP force plot can effectively clarify and explain model predictions for individual patients. The SHAP force plot for the XGBoost model is shown in **Figures 8C, D**. SHAP values represent the relevant predictive features of individual patients and the contribution of each feature to the prediction of the difficulty of LaTME surgery. Red indicates high surgical difficulty characteristics; blue indicates low surgical difficulty characteristics. The length of the arrow helps to achieve the size of the predicted effect. The longer the arrow, the greater the effect. **Figure 8C** shows a rectal cancer patient whose tumor height is 5.0cm, PNI is 39.1, angle 5 is 101.0°, pelvic inlet is 13.39cm, pelvic outlet is 7.62cm, mesorectal fat area is 14.44cm^2^ and sacrococcygeal distance is 13.40cm, with a Shapley value of 5.00(>base value). **Figure 8D** shows a rectal cancer patient whose tumor height is 5.0cm, PNI is 54.05, angle 5 is 97.3°, pelvic inlet is 12.18cm, pelvic outlet is 8.73cm and sacrococcygeal distance is 13.28cm, with a Shapley value of -2.94 (<base value). The advantage of this force plot is that it gives a clear combination of parameters that contribute greatly to the model.

## 4 Discussion

In our study, an accurate model was developed to predict the difficulty of rectal cancer surgery, and six machine learning prediction models were developed and evaluated. The prediction performance of XGBoost model is generally the best, AUC (0.855), F1 score (0.583), accuracy (0.841), sensitivity (0.538), specificity (0.92). However, LGBM has the highest specificity (0.93), LR has the highest sensitivity (0.731), and MLP has the highest F1 score (0.590). Seven core predictors of the difficulty of rectal surgery were determined by LASSO method, ten-fold cross-validation and multivariable logistic regression. The smaller the value of tumor height, PNI, pelvic inlet and pelvic outlet is, the higher the difficulty of operation is, while the higher the value of sacrococcygeal distance, mesorectal fat area and angle 5 is, the more difficult the operation is. Therefore, this study may be helpful to identify patients at risk of difficulty in operation. SHAP found that tumor height, pelvic inlet, sacrococcygeal distance, angle 5, PNI, mesorectal fat area and pelvic outlet, were ranked in order of importance related to surgical difficulty of LaTME.

It is well known that laparoscopic surgery for rectal cancer is considered technically difficult. Recent studies have shown that a variety of factors related to the difficulty of LaTME surgery, including doctors’ surgical skills, previous abdominal surgery history, preoperative radiotherapy, tumor height, body mass index (BMI), pelvic size, preoperative nutritional status and other factors can affect the difficulty of laparoscopic surgery (18, 23, 33–36). Actually, the definition of the difficulty of rectal surgery is actually vague. The definition of surgical difficulty should be a representative parameter, which can represent the factors related to the surgical results. In our study, we adopted the surgical difficulty classification criteria proposed by Escal (18) et al.: duration of surgery, estimated blood loss, conversion to open procedure, morbidity (grade II and III), use of transanal dissection and postoperative hospital stay, and slightly modified them. It makes sense to include both surgical and postoperative parameters in the criteria, as impaired surgical quality and variable postoperative course may increase local recurrence and impaired survival (37).

In our study, tumor height was the most important factor for surgical difficulty in LaTME, and this result is consistent with previous studies (38). Tumor height is one of the main factors in selecting surgical methods. The lower the tumor location, the more difficult transabdominal surgery is, and the more likely the surgeon is to choose laparoscopically assisted transsphincter plane ultra-low anterior rectal resection (35). In our study, univariable logistic regression showed that tumor height was associated with use of transanal dissection (P=0.009). The closer the tumor is to the anal verge, the greater the extent of dissection and exposure, and the more difficult the operation.

Our research shows that pelvic anatomy is the independent influencing factor affecting the difficulty of laparoscopic rectal cancer surgery. Pelvic measurement was originally used to evaluate the possibility of successful vaginal delivery (39). With the continuous development of laparoscopic technology, many colorectal experts are more and more interested in pelvic measurement in recent years. Pelvic measurement has been used to evaluate the difficulty of rectal cancer surgery, but the relationship between quantitative pelvic measurement and surgical difficulty has not been determined (18, 40–42), and even some studies have found that there is no relationship between pelvimetry and surgical difficulty (5, 43, 44). However, there are also some differences between our research and theirs. For example, Ogiso (5) et al. studied patients undergoing laparoscopic resection of rectal cancer, and the results showed that there was no correlation between pelvic parameter and operation time, but their study was based on only 50 cases. 626 patients who underwent laparoscopic rectal surgery were included in our study, and we used 20 pelvic measurement parameters based on MRI, including 8 longitudes, 11 angles, and 1 region. Multivariate logistic regression showed that pelvic inlet, pelvic outlet and sacrococcygeal distance were independent influencing factors for the difficulty of LaTME. This is partially consistent with previous findings. Multivariate analysis by Zhou (45) et al. showed that BMI, tumor height, lymph node metastasis, pelvic inlet, pelvic outlet, superior and inferior diameter of pubis, depth of sacrococcyx curvature, sacrococcyx-pubic angle and distance from pubic bone to coccyx were the main factors affecting operation time. By studying patients with rectal cancer receiving TaTME, Ferko (46) et al. found that the sharper the Angle 5, the more difficult the operation, and the worse the quality of TME. This is contrary to our results and may be due to different definitions of surgical difficulty and surgical methods. Laparoscopic surgery differs from other surgical techniques in its ability to access the pelvis, providing a multi-angle surgical field of view that is not achievable with open surgery. However, laparoscopic rectal cancer surgery presents greater challenges due to the deep anatomical position of the rectum within a narrow funnel-shaped pelvis, intricate surrounding tissue, and limited surgical space. Moreover, this procedure necessitates the use of rigid long-handled endoscopic instruments for complex operations such as cutting, separation, hemostasis, and anastomosis. These instruments differ significantly from traditional manual techniques and lack tactile feedback. Consequently, our study found that the narrow pelvic entrance and outlet, increased pelvic depth, and larger angle5 pose difficulties in terms of visual field visibility, accessibility to the operating area for LaTME in rectal cancer cases (47, 48), thereby increasing surgical complexity.

PNI is a protective factor to predict the difficulty of LaTME. The nutritional status of patients before operation is usually considered to be closely related to postoperative complications, such as postoperative anastomotic fistula, intestinal obstruction, ascites and so on (48, 49). In our study, PNI is related to conversion to open procedure, and low preoperative PNI is independently related to high difficulty of rectal surgery. However, Sun (23) et al. included 294 patients with locally advanced rectal cancer who underwent LaTME after preoperative radiotherapy and chemotherapy. It was found that preoperative AGR can predict the difficulty of rectal surgery after preoperative radiotherapy and chemotherapy. The difference is that the patients with preoperative radiotherapy and chemotherapy were excluded from our study. The PNI=49.5 ± 5.22 and AGR=1.52 (1.37~1.69) in our study were higher than those in their study PNI=46.0 ± 6.4 and AGR=1.3 ± 0.2. Therefore, preoperative radiotherapy and chemotherapy will damage the nutritional status of patients with rectal cancer. Often malnutrition and preoperative radiotherapy and chemotherapy are easy to cause tissue edema, fibrosis, extensive fog and exudate (47), which hinder tissue anatomy and increase the difficulty of operation. Unfortunately, it is not clear whether nutritional status will lead to different tissue responses to radiotherapy and chemotherapy. In addition, the mechanism of nutritional status predicting the difficulty of operation remains to be further discussed.

The current findings indicate that mesorectal fat area is considered an independent risk factor for surgical difficulty. In general, obesity can make rectal surgery more difficult (50, 51). The main reasons for these difficulties are dissection difficulties caused by the reduced relative space in the abdomen due to obesity, exposure problems (bowel layering, mesorectal volume) and the thickness of adipose tissue. In addition, the bulky mesentery is prone to tearing and bleeding. Lacerations resulting from mesenteric traction may result in unacceptable bleeding and thus clutter the surgical field. Unclear anatomy, intraoperative bleeding, intra-abdominal adhesions and intestinal perforation are common reasons for conversion to open surgery in obese patients (52). In addition, a recent meta-analysis (53) suggested that the incidence of anastomotic leakage, pulmonary events, and postoperative intestinal obstruction was significantly higher in the obese group, but this did not directly affect pathological safety. BMI represents the most common index describing overall obesity, and multiple studies have confirmed the negative impact of BMI on rectal surgery (5, 45, 54, 55). However, in our study, BMI was closely related to conversion to open procedure (p=0.037) and had no significant impact on surgical difficulty. This is because BMI may not accurately reflect changes in visceral fat distribution or overall obesity in the body. According to research, BMI is less sensitive, and for any given BMI value, there are large age, race, and gender differences in body fat percentage. For example, at the same BMI, Asians have higher body fat percentages than Caucasians (56). Therefore, BMI does not reflect the impact of obesity on laparoscopic rectal surgery, and mesorectal fat area may be a better indicator of the difficulty of laparoscopic rectal surgery.

Our research shows that the method of machine learning is feasible and has high accuracy. At present, because most prediction tools are developed in a linear and cumulative manner based on the interaction of variables (57), their clinical applicability is limited and their predictive ability is poor. However, the surgical complexity of LaTME is multifactorial, and the relationship between surgical difficulty and influencing factors is not entirely linear. In recent years, machine learning algorithms have been extensively utilized in the field of medicine and have emerged as a powerful tool for addressing numerous clinical predictions. Machine learning algorithms can effectively overcome the limitations of traditional methods and serve as a more accurate and non-linear approach to predicting patient prognosis (58, 59). In fact, previous studies have developed models that use machine learning techniques to predict the difficulty of rectal cancer surgery. For example, Lv (60)et al. established a blood loss and resection duration (BLADE) scoring system, and used RF algorithm to establish a preoperative prediction model of BLADE score. Our research focuses on early identification of predictors that affect the difficulty of LaTME surgery. In addition, many machine learning models are black-box models, lack of variable relationship analysis in clinical application, and this problem also exists in our model. Therefore, we introduce SHAP to explain the output prediction model, which provides a convincing explanation for the relationship between nonlinear variables (61). As an interpretable omnipotent method of the model, SHAP can be used for global and local interpretation. SHAP analysis can guide clinicians to pay attention to target variables in patients with high surgical difficulty, which is more beneficial to the evaluation of patients before operation.

The results of the current study have several clinical implications. First, for patients with poor preoperative nutritional status, the patient’s albumin level should be improved first before LaTME is performed. Second, for patients with rectal cancer in a difficult pelvis, it can help improve patient-physician communication by informing patients of possible perioperative risks and complications and selecting an appropriate surgical approach (e.g., open, laparoscopic, robotic, or transanal Operation). Finally, early career surgeons can select appropriate cases during the learning process, and patients with difficult pelvises can be referred to more specialized doctors and experienced surgeons to improve surgical quality and minimize the risk of complications and adverse consequences due to lack of experience. Other surgeons can collect clinicopathological and MRI pelvimetry from their patients and input them into our XGBoost machine learning models to get accurate clinical predictions. The SHAP force plot can be output to show the influence of each variable on the difficulty of LaTME surgery.

This study has some limitations. On the one hand, this is a single-center retrospective study, there is inevitable selection bias, difficult surgical risk factors and predictive models can be widely used in patients with rectal cancer, need to be further studied and verified. On the other hand, this study did not explore the survival and prognosis of the two groups of patients, and the data are limited. Therefore, it is necessary to conduct prospective randomized studies with larger samples and longer follow-up periods to simulate the interaction between variables. In addition, we only use 2D MRI pelvic measurements, excluding 3D features. 3D pelvic measurements should be further evaluated to better explore the relationship between pelvic features and surgical difficulty.

## 5 Conclusion

In our study, we developed a model based on the XGBoost machine learning algorithm to predict the surgical difficulty of LaTME. The model has good prediction accuracy and clinical practicability, which is helpful for surgeons to identify patients with high surgical difficulty as early as possible. The model identifies tumor height, PNI, pelvic inlet, pelvic outlet, sacrococcygeal distance, mesorectal fat area and angle 5 as independent influencing factors.

## Author contributions

MY: Writing – original draft, Data curation, Software, Validation, Methodology. ZY: Conceptualization, Investigation, Writing – original draft, Data curation, Validation. RL: Data curation, Investigation, Writing – original draft. BS: Data curation, Methodology, Writing – original draft. DW: Conceptualization, Supervision, Validation, Writing – review & editing, Visualization. XD: Conceptualization, Resources, Supervision, Validation, Writing – review & editing.
