# Supplementary material for: Interpretable machine learning model to predict surgical difficulty in laparoscopic resection for rectal cancer
Source: Front Oncol. 2024 Feb 6;14:1337219. doi: 10.3389/fonc.2024.1337219 (PMC10878416; doi:10.3389/fonc.2024.1337219)
Supplement: Supplementary file 1 [file Table_1.docx]

| **Supplemental Table 1. Univariable logistic regression analyses of associations between all factors and surgical difficulty criteria** | | | | | | | | | | | | |
| --- | --- | --- | --- | --- | --- | --- | --- | --- | --- | --- | --- | --- |
|  | Duration of  surgery | | Estimated  blood loss | | Conversion to open procedure | | Morbidity  (grade II and III) | | Use of  transanal dissection | | Postoperative  hospital stay | |
|  | OR (95%CI) | *P* | OR (95%CI) | *P* | OR (95%CI) | *P* | OR (95%CI) | *P* | OR (95%CI) | *P* | OR (95%CI) | *P* |
| **Baseline characteristics** |  |  |  |  |  |  |  |  |  |  |  |  |
| Gender |  | 0.738 |  | 0.726 |  | 0.477 |  | 0.161 |  | 0.656 |  | 0.089 |
| male | 1 (reference) |  | 1 (reference) |  | 1 (reference) |  | 1 (reference) |  | 1 (reference) |  | 1 (reference) |  |
| femal | 0.941  (0.661,1.340) | | 1.065  (0.749,1.515) |  | 1.149  (0.804,1.641) |  | 0.776  (0.545,1.106) |  | 1.092  (0.742,1.605) |  | 0.720  (0.493,1.052) |  |
| Age(year) | 1.000  (0.985,1.015) | 0.994 | 0.999  (0.984,1.014) | 0.908 | 0.995  (0.981,1.010) | 0.548 | 1.011  (0.995,1.026) | 0.171 | 1.005  (0.988,1.021) | 0.586 | 0.991  (0.976,1.006) | 0.241 |
| BMI (kg/m²) | 1.022  (0.967,1.080) | 0.434 | 1.012  (0.957,1.069) | 0.685 | 0.940  (0.888,0.996) | **0.037** | 1.030  (0.975,1.088) | 0.294 | 0.998  (0.939,1.061) | 0.950 | 0.983  (0.927,1.042) | 0.565 |
| Tumor height(cm) | 1.014  (0.959,1.073) | 0.615 | 0.981  (0.927,1.038) | 0.503 | 1.039  (0.982,1.100) | 0.184 | 0.991  (0.938,1.048) | 0.754 | 0.947  (0.890,1.008) | **0.009** | 0.976  (0.920,1.036) | 0.422 |
| **Hematology**  **nutritional indicators** |  |  |  |  |  |  |  |  |  |  |  |  |
| AGR | 0.548  (0.284,1.055) | 0.072 | 1.127  (0.590,2.154) | 0.717 | 1.587  (0.822,3.064) | 0.169 | 1.047  (0.552,1.986) | 0.888 | 0.698  (0.342,1.427) | 0.325 | 0.758  (0.383,1.500) | 0.426 |
| PNI | 0.996  (0.963,1.029) | 0.807 | 1.018  (0.985,1.053) | 0.294 | 1.036  (1.001,1.072) | **0.043** | 0.998  (0.966,1.032) | 0.916 | 0.998  (0.962,1.035) | 0.897 | 1.010  (0.975,1.046) | 0.572 |
| **Pathological stage** |  |  |  |  |  |  |  |  |  |  | 0.758  (0.383,1.500) | 0.426 |
| Pathological T stage |  | 0.290 |  | 0.776 |  | **0.049** |  | 0.314 |  | 0.939 |  | 0.918 |
| T1 | 1 (reference) |  | 1 (reference) |  | 1 (reference) |  | 1 (reference) |  | 1 (reference) |  | 1 (reference) |  |
| T2 | 0.665  (0.120,3.671) | 0.639 | 2.696  (0.311,23.350) | 0.368 | 0.515  (0.341,0.779) | 0.452 | 0.833  (0.152,4.567) | 0.834 | 1.595  (0.182,13.983) | 0.673 | 0.705  (0.128,3.886) | 0.688 |
| T3 | 1.108  (0.212,5.777) | 0.903 | 2.400  (0.286,20.124) | 0.420 | 0.371  (0.303,0.455) | 0.245 | 1.053  (0.202,5.493) | 0.951 | 1.668  (0.198,14.009) | 0.638 | 0.831  (0.159,4.340) | 0.826 |
| T4 | 0.972  (0.169,5.607) | 0.975 | 2.108  (0.231,19.204) | 0.508 | 0.250  (0.125,0.500) | **0.037** | 1.667  (0.294,9.445) | 0.564 | 1.895  (0.207,17.345) | 0.572 | 0.878  (0.152,5.092) | 0.885 |
| Pathological N stage |  | 0.548 |  | 0.836 |  | 0.17 |  | 0.615 |  | 0.582 |  | 0.341 |
| N0 | 1 (reference) |  | 1 (reference) |  | 1 (reference) |  | 1 (reference) |  | 1 (reference) |  | 1 (reference) |  |
| N1 | 0.964  (0.635,1.463) | 0.862 | 1.131  (0.751,1.704) | 0.555 | 0.962  (0.637,1.452) | 0.853 | 1.114  (0.740,1.677) | 0.605 | 1.267  (0.812,1.977) | 0.298 | 1.284  (0.842,1.959) | 0.246 |
| N2 | 1.237  (0.803,1.903) | 0.335 | 1.022  (0.656,1.594) | 0.922 | 0.640  (0.398,1.029) | 0.065 | 1.237  (0.0803,1.903) | 0.335 | 1.090  (0.669,1.775) | 0.730 | 0.892  (0.552,1440) | 0.64 |
| Pathological TNM stage |  | 0.440 |  | 0.892 |  | 0.345 |  | 0.62 |  | 0.102 |  | 0.808 |
| Ⅰ | 1 (reference) |  | 1 (reference) |  | 1 (reference) |  | 1 (reference) |  | 1 (reference) |  | 1 (reference) |  |
| Ⅱ | 1.450  (0.802,2.620) | 0.219 | 0.925  (0.528,1.169) | 0.785 | 0.688  (0.399,1.186) | 0.178 | 0.871  (0.500,1.516) | 0.624 | 1.931  (0.932,4.000) | 0.077 | 1.185  (0.652,2.155) | 0.578 |
| Ⅲ | 1.429  (0.799,2.556) | 0.228 | 1.011  (0.586,1.744) | 0.970 | 0.688  (0.405,1.171) | 0.168 | 1.046  (0.611,1.791) | 0.871 | 2.179  (1.067,4.450) | **0.033** | 1.070  (0.594,1.928) | 0.821 |
| **MRI pelvimetry** |  |  |  |  |  |  |  |  |  |  |  |  |
| Pelvic inlet(cm) | 0.966  (0.821,1.136) | 0.673 | 1.026  (0.872,1.208) | 0.756 | 1.001  (0.849,1.180) | 0.991 | 0.960  (0.817,1.127) | 0.617 | 0.977  (0.818,1.168) | 0.799 | 0.953  (0.803,1.130) | 0.577 |
| Middle pelvis(cm) | 0.901  (0.756,1.073) | 0.242 | 0.980  (0.823,1.167) | 0.820 | 0.936  (0.783,1.118) | 0.463 | 0.977  (0.822,1.162) | 0.795 | 1.036  (0.856,1.225) | 0.713 | 0.892  (0.741,1.073) | 0.225 |
| Pelvic outlet(cm) | 0.904  (0.744,1.098) | 0.308 | 0.996  (0.820,1.210) | 0.969 | 1.042  (0.856,1.269) | 0.681 | 1.007  (0.831,1.220) | 0.945 | 0.942  (0.760,1.166) | 0.580 | 0.970  (0.791,1.189) | 0.768 |
| Interischial distance (cm) | 1.025  (0.975,1.077) | 0.329 | 0.980  (0.908,1.058) | 0.605 | 0.946  (0.824,1.085) | 0.425 | 0.967  (0.871,1.073) | 0.528 | 0.974  (0.877,1.081) | 0.616 | 0.931  (0.803,1.080) | 0.346 |
| Intertuberous distance (cm) | 0.903  (0.817,0.997) | **0.043** | 0.953  (0.864,1.051) | 0.334 | 0.962  (0.871,1.062) | 0.445 | 0.956  (0.868,1.053) | 0.364 | 0.928  (0.833,1.034) | 0.176 | 0.926  (0.835,1.027) | 0.147 |
| Pubic symphysis height(cm) | 1.053  (0.794,1.397) | 0.719 | 1.012  (0.762,1.344) | 0.934 | 0.837  (0.626,1.120) | 0.232 | 0.747  (0.561,0.996) | **0.047** | 1.221  (0.896,1.665) | 0.206 | 0.982  (0.729,1.323) | 0.906 |
| Sacrococcygeal distance(cm) | 0.971  (0.842,1.120) | 0.687 | 0.983  (0.852,1.135) | 0.816 | 1.079  (0.932,1.249) | 0.311 | 0.982  (0.852,1.131) | 0.798 | 0.983  (0.840,1.150) | 0.827 | 1.051  (0.904,1.223) | 0.516 |
| Internal diameter of sacrum and pubis(cm) | 0.929  (0.798,1.082) | 0.346 | 0.998  (0.856,1.162) | 0.976 | 1.251  (1.068,1.464) | **0.005** | 0.945  (0.813,1.099) | 0.465 | 0.914  (0.773,1.081) | 0.296 | 0.952  (0.811,1.118) | 0.55 |
| Mesorectal fat area(cm2) | 0.976  (0.950,1.003) | 0.081 | 0.958  (0.932,0.98) | **0.003** | 1.002  (0.975,1.030) | 0.867 | 0.994  (0.967,1.020) | 0.632 | 0.991  (0.963,1.021) | 0.568 | 0.994  (0.996,1.022) | 0.667 |
| Angle 1 | 1.008  (0.995,1.021) | 0.231 | 1.008  (0.995,1.021) | 0.221 | 0.995  (0.983,1.008) | 0.995 | 1.006  (0.994,1.019) | 0.324 | 0.991  (0.978,1.004) | 0.186 | 1.007  (0.994,1.020) | 0.301 |
| Angle 2 | 0.999  (0.983,1.015) | 0.889 | 1.003  (0.987,1.019) | 0.687 | 1.009  (0.993,1.025) | 0.295 | 0.989  (0.973,1.004) | 0.156 | 0.997  (0.980,1015) | 0.764 | 0.998  (0.981,1.015) | 0.794 |
| Angle 3 | 1.004  (0.986,1.022) | 0.693 | 0.997  (0.979,1.015) | 0.728 | 0.987  (0.970,1.005) | 0.155 | 1.032  (1.013,1.052) | **0.001** | 1.001  (0.981,1.021) | 0.933 | 1.015  (0.995,1.035) | 0.133 |
| Angle 4 | 0.994  (0.977,1.011) | 0.494 | 0.994  (0.977,1.011) | 0.481 | 0.995  (0.978,1.012) | 0.549 | 0.997  (0.980,1.014) | 0.711 | 1.014  (0.996,1.033) | 0.133 | 0.993  (0.976,1.011) | 0.446 |
| Angle 5 | 0.992  (0.977,1.007) | 0.307 | 0.993  (0.977,1.008) | 0.36 | 1.011  (0.996,1.027) | 0.147 | 0.979  (0.964,0.996) | **0.012** | 1.004  (0.988,1.021) | 0.62 | 0.987  (0.970,1.003) | 0.115 |
| Sacrococcygeal–pubic angle | 0.992  (0.972,1.011) | 0.392 | 0.985  (0.965,1.004) | 0.122 | 1.003  (0.984,1.023) | 0.763 | 1.003  (0.984,1.023) | 0.727 | 1.008  (0.987,1.029) | 0.474 | 0.996  (0.976,1.016) | 0.697 |
| Angle T1 | 1.007  (0.998,1.015) | 0.121 | 0.998  (0.989,1.007) | 0.648 | 0.994  (0.984,1.003) | 0.165 | 1.011  (1.003,1.019) | **0.008** | 0.989  (0.979,0.999) | **0.039** | 1.001  (0.992,1.010) | 0.91 |
| Angle T2 | 1.004  (0.998,1.009) | 0.177 | 0.999  (0.993,1.005) | 0.762 | 0.996  (0.990,1.001) | 0.143 | 1.006  (1.001,1.012) | **0.025** | 0.988  (0.982,0.995) | **0.001** | 0.996  (0.990,1.002) | 0.218 |
| Angle T3 | 0.996  (0.992,1.000) | 0.07 | 1.000  (0.996,1.004) | 0.931 | 1.003  (0.999,1.007) | 0.175 | 0.994  (0.990,0.998) | **0.006** | 1.010  (1.005,1.014) | **<0.001** | 1.001  (0.997,1.006) | 0.537 |
| Angle T4 | 1.025  (0.998,1.053) | 0.068 | 1.020  (0.993,1.048) | 0.146 | 0.986  (0.959,1.014) | 0.317 | 1.012  (0.985,1.039) | 0.394 | 1.000  (0.971,1.030) | 0.994 | 1.051  (1.021,1.081) | **0.001** |
| Angle T5 | 0.998  (0.988,1.007) | 0.631 | 1.003  (0.994,1.012) | 0.525 | 1.005  (0.997,1.014) | 0.227 | 0.997  (0.987,1.006) | 0.499 | 0.989  (0.977,1.001) | 0.084 | 0.996  (0.986,1.007) | 0.485 |

OR, odds ratio; CI, confidence interval; IQR, interquartile range; SD, standard deviation; BMI, body mass index; AGR, albumin to globulin ratio; PNI, prognostic nutrition index; The bold values P <0.05.
