# Supplementary material for: Interpretable machine learning model to predict surgical difficulty in laparoscopic resection for rectal cancer
Source: Front Oncol. 2024 Feb 6;14:1337219. doi: 10.3389/fonc.2024.1337219 (PMC10878416; doi:10.3389/fonc.2024.1337219)
Supplement: Supplementary file 2 [file Table_2.docx]

| **Supplemental Table 2. Comparison of clinical features and MRI pelvimetry between training and test group** | | | |
| --- | --- | --- | --- |
| **Variables** | **Training group**  **(N=500)** | **Test group**  **(N = 126)** | ***P*** |
| **Baseline characteristics** |  |  |  |
| Gender (%) |  |  | 0.955 |
| Male | 299(59.8%) | 75(59.52%) |  |
| Female | 201(40.2%) | 51(40.48%) |  |
| Age (median [IQR]，year) | 64(56,71) | 64(57,70) | 0.748 |
| BMI (median [IQR]，kg/m²) | 23.62(21,61,25.39) | 23.88(21.12,25.84) | 0.655 |
| Tumor height  (median [IQR]，cm) | 9.0(7.0,12.0) | 9.3(7.0,12.0) | 0.735 |
| **Hematology nutritional indicators** |  |  |  |
| AGR (median [IQR]) | 1.52(1.38,1.69) | 1.54(1.32,1.73) | 0.656 |
| PNI (mean [SD]) | 49.44(5.34) | 49.72(4.69) | 0.590 |
| **Pathological stage** |  |  |  |
| Pathological T stage (%) |  |  | 0.610 |
| T1 | 6(1.2%) | 1(0.79%) |  |
| T2 | 81(16.2%) | 19(15.08%) |  |
| T3 | 374(74.8) | 95(75.40%) |  |
| T4 | 39(7.8%) | 11(8.73%) |  |
| Pathological N stage (%) |  |  | 0.202 |
| N0 | 261(52.2%) | 59(46.83%) |  |
| N1 | 134(26.8%) | 34(26.98%) |  |
| N2 | 105(21%) | 33(26.19%) |  |
| Pathological TNM stage (%) |  |  | 0.166 |
| Ⅰ | 66(13.2%) | 13(10.32%) |  |
| Ⅱ | 201(40.2%) | 46(36.51%) |  |
| Ⅲ | 233(46.6%) | 67(53.17%) |  |
| **MRI pelvimetry** |  |  |  |
| Pelvic inlet  (mean [SD], cm) | 11.75(1.06) | 11.68(1.08) | 0.547 |
| Middle pelvis  (mean [SD], cm) | 12.55(1.00) | 12.57(0.95) | 0.850 |
| pelvic outlet  (mean [SD], cm) | 8.76(0.91) | 8.85(0.80) | 0.293 |
| Interischial distance (median [IQR], cm) | 9.72(8.97,10.63) | 9.89（8.66,10.68） | 0.985 |
| Intertuberous distance (median [IQR], cm) | 9.97(8.77,11.21) | 10.01(8.81,11.09) | 0.720 |
| Pubic symphysis height (median [IQR], cm) | 4.72(4.27,5.13) | 4.80(4.26,5.19) | 0.379 |
| Sacrococcygeal distance (median [IQR], cm) | 12.60(11.68,13.33) | 12.61(11.63,13.47) | 0.900 |
| Internal diameter of sacrum and pubis (mean [SD], cm) | 12.88(1.13) | 12.68(1.17) | 0.190 |
| Mesorectal fat area (median [IQR], cm²) | 16.29(11.52,21.36) | 18.75(11.93,22.44) | 0.083 |
| Angle 1 (median [IQR], °) | 116.6(107.8,124.6) | 114.4(104.4,123.1) | 0.169 |
| Angle 2 (mean [SD], °) | 108.3(10.79) | 108.3(11.4) | 0.993 |
| Angle 3 (median [IQR], °) | 127.05(122.2，132.4) | 127.3(122.2,135.0) | 0.557 |
| Angle 4 (median [IQR], °) | 88.4(82.0,96.0) | 90.3(84.1,98.0) | 0.100 |
| Angle 5 (median [IQR], °) | 98.5(93.8,103.6) | 98.2(92.8,105.2) | 0.757 |
| Sacrococcygeal–pubic angle (median [IQR],°） | 47.0(41.2,51.8) | 46.8(39.4,53.5) | 0.816 |
| Angle T1(median [IQR], °) | 55.4(46.9,70.4) | 51.5(46.3,61.6) | **0.015** |
| Angle T2 (median [IQR], °) | 79.5(55.8,101.2) | 80.8(59.5,105.3) | 0.916 |
| Angle T3 (median [IQR], °) | 107.5(78.1,140.5) | 122.1(90.9,149.1) | **0.003** |
| Angle T4 (median [IQR], °) | 27.1(23.2,31.3) | 26.2(22.0,29.7) | 0.061 |
| Angle T5 (median [IQR], °) | 73.4(67.4,81.1) | 68.4(61.77,74.5) | **<0.001** |
| **Surgical difficulty** |  |  |  |
| Duration of surgery (median [IQR], min) | 181.5(140.0,240,0) | 181.5(148.0,235.0) | 0.797 |
| Blood loss (median [IQR], ml) | 100(50,200) | 100(50,200) | 0.926 |
| Postoperative hospital stays  (median [IQR], day) | 10(8,12) | 9(8,14) | 0.777 |
| Morbidity (grade II and III) (yes/no, %) | 150/350(30/70) | 36/90(28.3/71.7) | 0.754 |
| Use of transanal dissection (yes/no, %) | 98/402(19.6/80.4) | 38/88(30.2/69.8) | 0.08 |
| Conversion to open procedure (yes/no, %) | 139/361(27.8/71.2) | 32(25.4/74.6) | 0.588 |

IQR, interquartile range; SD, standard deviation; BMI, body mass index; AGR, albumin to globulin ratio; PNI, prognostic nutrition index; The bold values P <0.05.
