# Supplementary material for: Interpretable machine learning model to predict surgical difficulty in laparoscopic resection for rectal cancer
Source: Front Oncol. 2024 Feb 6;14:1337219. doi: 10.3389/fonc.2024.1337219 (PMC10878416; doi:10.3389/fonc.2024.1337219)
Supplement: Supplementary file 3 [file Table_3.docx]

**Supplemental Table 3.** **Full super-parameters of technique**

| **Algorithm** | **Parameter** |
| --- | --- |
| LGBM | Colsample_bytree=0.7, learning_rate=0.1, max_depth= -1,  n_estimators= 100, num_leaves=31, subsample=0.5 |
| XGBoost | Colsample_bytree: =0.5, gamma=0, learning_rate=0.01,  max_depth = 7, n_estimators=500 |
| CatBoost | depth= 5, learning_rate=0.05, subsample=0.8 |
| LR | C= 100, max_iter= 100, penalty=l2, solver=liblinear |
| RF | bootstrap=False, max_depth=None, max_features=log2, min_samples_leaf=1, min_samples_split=2, n_estimators=200 |
| MLP | activation=tanh, alpha=0.05, hidden_layer_sizes=50, learning_rate=constant, solver=adam |

LR, logistic regression; LGBM, light gradient boosting machine; CatBoost, categorical boosting; MLP, multilayer perceptron; RF, random forests; XGBoost, extreme gradient boost
